# Applying a validated scoring rubric to pre-clerkship medical students’ standardized patient notes: a pilot study

**DOI:** 10.1186/s12909-023-04424-9

**Published:** 2023-07-13

**Authors:** Benjamin D. Gallagher, Michael L. Green, Jaideep S. Talwalkar

**Affiliations:** 1 Department of Internal Medicine, Yale School of Medicine, 789 Howard Avenue, New Haven, CT USA; 2 Department of Pediatrics, Yale School of Medicine, New Haven, CT USA

**Keywords:** Undergraduate medical education, Clinical reasoning, Clinical assessment, OSCE, Scoring rubric

## Abstract

**Background:**

With the elimination in 2021 of the United States Medical Licensing Examination Step 2 Clinical Skills test, it is incumbent upon U.S. medical schools to develop local validated assessments of clinical reasoning. While much attention has been paid to summative exams for graduating students, formative exams for pre-clerkship students have not been well studied.

**Methods:**

We applied the University of Illinois at Chicago College of Medicine (UIC-COM) Patient Note (PN) Scoring Rubric to templated PNs written by 103 pre-clerkship students for two cases in an objective structured clinical examination (OSCE) at the Yale School of Medicine. The rubric consists of four section scores (Documentation, Differential Diagnosis, Justification, and Workup, each scored 1 to 4) and a composite score (scaled 23 to 100). We calculated item discrimination for each section score and Cronbach’s alpha for each case. We surveyed students about their experience writing the templated PN.

**Results:**

Mean Documentation, Differential Diagnosis, Justification, Workup, and composite scores for case A were 2.16, 1.80, 1.65, 2.29, and 47.67, respectively. For case B, the scores were 2.13, 1.21, 1.60, 1.67, and 40.54, respectively. Item discrimination ranged from 0.41 to 0.80. Cronbach’s alpha for cases A and B was 0.48 and 0.25, respectively. A majority of the students felt that the exercise was useful and appropriate to their level of training.

**Conclusions:**

Despite performing poorly, pre-clerkship students found the note-writing task beneficial. Reliability of the scoring rubric was suboptimal, and modifications are needed to make this exercise a suitable measure of clinical reasoning.

**Supplementary Information:**

The online version contains supplementary material available at 10.1186/s12909-023-04424-9.

## Background

Assessing clinical reasoning skills is a key task for teachers of medical students [1]. The United States Medical Licensing Examination (USMLE) Step 2 Clinical Skills (CS) exam was designed to measure history-taking, physical examination, clinical reasoning, and communication skills among graduating medical students to ensure that they are ready for residency training. This exam, which consisted of timed standardized patient (SP) encounters, was postponed in March 2020 due to the COVID-19 pandemic and then discontinued permanently in January 2021. As a result, the responsibility of evaluating whether graduating students are competent diagnostic reasoners has been shifted entirely to individual medical schools. There is therefore a pressing need for assessments of clinical reasoning with defensible validity evidence that can be administered locally by medical schools [2].

In recent decades, many medical schools have developed objective structured clinical examinations (OSCEs) to assess clinical skills and prepare senior students for USMLE Step 2 CS [3]. One component of these OSCEs is a templated patient note (PN), in which students write a brief history and physical exam, list a ranked differential diagnosis with justification for each item, and propose a diagnostic workup. Unlike open-ended PNs that students more commonly submit to their preceptors, these templated PNs are free of interference from outside resources (e.g. medical textbooks, online search tools), making them a unique “point of care” test of clinical reasoning [4]. The University of Illinois at Chicago College of Medicine (UIC-COM) has created a rubric for scoring templated PNs and published validity evidence in senior students from their own institution [5–7] and from seven medical schools using five shared cases [8]. PN scores using this rubric were shown to correlate with core clinical rotation performance at another institution [4].

Clinical reasoning is a skill that grows over time, and, in this new post-Step 2 CS era, it will be important to develop formative assessments of clinical reasoning for pre-clerkship students to track progress toward graduation competency benchmarks and identify students in need of remediation [9]. Many medical schools already hold OSCEs in the first and/or second year, but to our knowledge there are no validated rubrics for scoring templated PNs written by pre-clerkship students. As a first step toward generating validity evidence for the UIC-COM PN Scoring Rubric in this new student population, we piloted the rubric in templated PNs written by students at the Yale School of Medicine (YSM) for their pre-clerkship OSCE, and calculated measures of internal consistency reliability. We also surveyed students about their experience writing the templated PN.

## Methods

### Yale School of Medicine (YSM) pre-clerkship OSCE

In November 2020, the YSM pre-clerkship OSCE was held for students in their third semester of medical school (i.e. the first half of the second year). In the first three semesters, students meet in small groups with longitudinal preceptors to practice the medical interview, physical exam, and note-writing, in addition to learning about these skills in large group didactics. During the OSCE, students are given 45 min to conduct a full history and physical exam and share their assessment and plan with the SP at two stations (hereafter referred to as cases A and B). Cases A and B were written and reviewed by a committee of content and educational experts from YSM and the University of Connecticut School of Medicine, adhering to best practices of case writing, and are reviewed and modified every year as necessary [3]. Both cases cover subject matter to which students had already been exposed in their pre-clerkship coursework. Due to COVID-19 restrictions, in 2020 the students interacted with the SPs via video conference and examined mannequins serving as surrogates for the SPs. SPs evaluate students on the completeness of their histories and physicals and on their communication skills. Students receive these SP scores and later review video recordings of the SP encounters with clinical faculty. A minimum passing score is required on the history, physical exam, and communication skills sections. Students who fail to meet this standard are remediated.

Prior to the implementation of this project, students were tasked with submitting only an open-ended PN for the second case of the OSCE within 48 h of completion. Because students are randomly assigned to complete case A or B first, approximately 50% write notes for cases A and B, respectively. Students are free to use outside resources to formulate their assessment and plan. Senior medical students review the open-ended PNs and provide unstructured feedback (i.e. there is no numerical score). Open-ended PN completion is required but does not count toward the OSCE score.

**Fig. 1:**
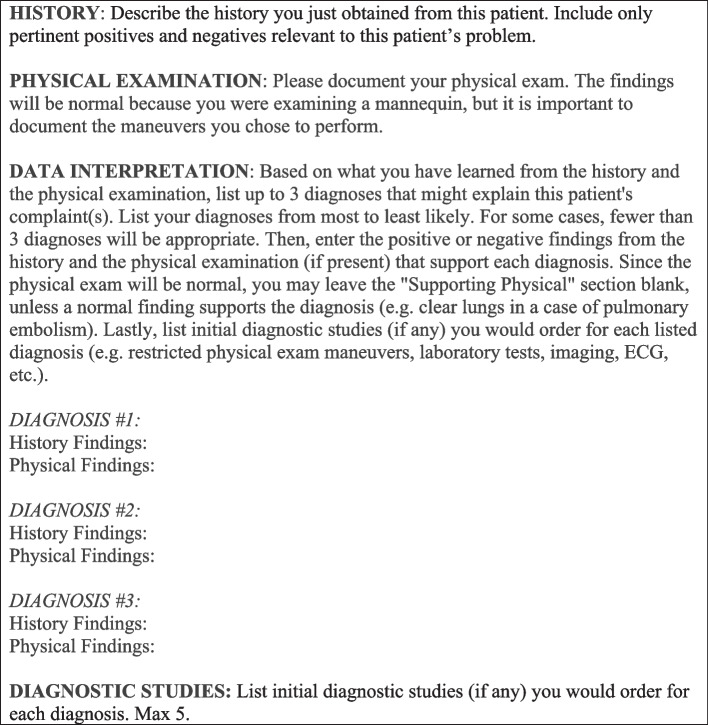
Patient note template used in an OSCE for pre-clerkship medical students at the Yale School of Medicine, 2020 Abbreviations: OSCE: objective structured clinical examination

For this project, students still completed an open-ended PN on the second case, but before doing so, we also asked them to write a templated PN in the style of the USMLE Step 2 CS exam (Fig. 1), to be completed during a 10-min break before receiving feedback from the SP. The scoring of the templated PN was likewise used for formative purposes only.

### Participants

One hundred three second-year students (the entire class) completed the OSCE. Fifty-one students completed a templated PN for case A, and 52 students for case B. Prior to taking part in the OSCE, students received an in-class announcement and follow-up email explaining the purpose of the study. Students were given the opportunity to opt out anonymously of having their data included in the study while still participating in the OSCE; none chose to do so.

### Scoring rubric

We used the UIC-COM PN Scoring Rubric to score the templated PNs (see Table 1 in Park et al*.*) [8]. The rubric contains four sections: Documentation, Differential Diagnosis, Justification, and Workup. Each section is scored on a scale of 1 to 4, with higher scores indicating higher performance. To generate a composite score with a maximum of 100 points, the Documentation, Differential Diagnosis, and Justification sections are each worth 30 points, and the Workup section is worth 10 points. Each section score level from the 1 to 4 scale is worth 25% of the maximum number of points for that section (e.g. 7 points for a Documentation score of 1, 15 points for a score of 2, 23 points for a score of 3, and 30 points for a score of 4).

We reviewed the case materials for the SP scenarios and collaborated on writing an exemplar Documentation section for each case. We shared these exemplars with eight general internists and asked them to generate differential diagnoses. From these submissions we developed a consensus Differential Diagnosis section for each case. One author (B.D.G.) then wrote exemplar Justification and Workup sections, which were reviewed and modified by the other authors until consensus was achieved. See Appendices A and B for the complete exemplar notes for cases A and B, respectively. One of us (B.D.G.) scored all of the students’ templated PNs using the exemplar notes; scoring took approximately 3 to 5 min per note. Another author (M.L.G.) scored five randomly selected students’ templated PNs for case A and five for case B to determine inter-rater reliability. Exact agreement for all unweighted templated PN section scores (40 total) was 60%, kappa (SE) was 0.40 (0.11), and quadratically weighted kappa (SE) was 0.47 (0.20).

After scoring was completed (several weeks after the OSCE was held), we shared with the students their templated PNs, section and composite scores, descriptive statistics for the class overall, the exemplar notes, and the UIC-COM PN Scoring Rubric.

### Survey

We developed a post-OSCE survey (Appendix C) regarding the following constructs: educational utility of the templated PN exercise, difficulty of the templated PN vs. open-ended PN, and preference for the templated PN vs. open-ended PN. After we drafted the survey items, they were reviewed and revised by an educational psychologist to demonstrate content validity. Because this was a pilot study, we did not collect reliability data regarding the survey. Three days after completing the templated PN, students were given the option of completing the survey; eighty (78%) did so.

### Statistical analyses

We used descriptive statistics to report templated PN section and composite scores for each case. We calculated two measures of internal consistency reliability: item discrimination (Pearson correlation coefficient between each section score and the composite score) and Cronbach’s alpha. Analyses were performed using SPSS (version 28.0, IBM Corp., Armonk, NY).

### Institutional approval

This study was declared exempt by the YSM Institutional Review Board. It was approved by the YSM Committee to Review Student Participation in Research.

## Results

The section and composite scores and item discriminations for cases A and B are shown in Table 1. The mean Documentation, Differential Diagnosis, Justification, Workup, and Composite Scores for Case A were 2.16, 1.80, 1.65, 2.29, and 47.67, respectively, and for Case B were 2.13, 1.21, 1.60, 1.67, and 40.54, respectively. Item discrimination ranged from 0.41 to 0.80 for case A and from 0.46 to 0.64 for case B. Cronbach’s alpha for cases A and B was 0.48 and 0.25, respectively. By comparison, in one multisite study of nearly 1,000 senior students, the mean Documentation, Differential Diagnosis, Justification, Workup, and Composite Scores across five cases were 2.83, 3.16, 2.94, 3.13, and 75, respectively [8]. In the multisite study, item discrimination across five cases ranged from 0.19 to 0.34, and Cronbach’s alpha was 0.46 [8].

**Table 1:** Patient note scores and item discrimination for case A (*n* = 51), and case B (*n* = 52)

	**Documentation**	**Differential Diagnosis**	**Justification**	**Workup**	**Composite Score**
*Case A*					
**Mean (SD)**	2.16 (0.54)	1.80 (0.85)	1.65 (0.66)	2.29 (0.88)	47.67 (12.42)
**Item discrimination**	0.43	0.80	0.78	0.41	N/A
*Case B*					
**Mean (SD)**	2.13 (0.82)	1.21 (0.41)	1.60 (0.66)	1.67 (0.62)	40.54 (9.37)
**Item discrimination**	0.51	0.46	0.64	0.58	N/A

We observed that not all students completed all the templated PN sections. For case A, completion rates for the Documentation, Differential Diagnosis, Justification, and Workup sections were 100%, 92%, 86%, and 82%, respectively. For case B, completion rates were 100%, 90%, 85%, and 62%. Because the lowest score on the rubric for each section (1 point out of 4) does not distinguish between incomplete and poor responses, we rescored all templated PN sections with a score of 0 representing an incomplete response. However, this resulted in only small changes in the section and composite scores and in the measures of internal consistency reliability (data not shown).

In the post-OSCE survey, 71% of students somewhat or strongly agreed, on a 5-point Likert scale, that writing the templated PN was useful for writing a history and physical. Seventy-one percent found it useful for developing a differential diagnosis, 69% found it useful for justifying a differential diagnosis, and 45% found it useful for devising a treatment plan. Eighty-seven percent of students somewhat or strongly agreed that the OSCE was appropriate for their level of training. Seventy-one percent somewhat or strongly disagreed that knowing they had to write the templated PN negatively affected their ability to engage with the SP. When asked about the relative levels of difficulty of the templated and open-ended PNs, 31% found the templated PN more difficult, 19% of students found the open-ended PN more difficult, and 50% found no difference. Among those reporting that the templated PN was more difficult, the time limit and lack of familiarity with the format were frequently cited as factors. Regarding which PN type they preferred, 41% preferred the templated PN, 21% preferred the open-ended PN, and 38% expressed no preference. Those who preferred the templated PN found the structure helpful, whereas those who preferred the open-ended PN preferred it because they were more familiar with the format, felt less constrained in expressing their thoughts, and did not have a time limit.

## Discussion

In this study, we found high student satisfaction but poor student performance and low reliability of a rubric developed by Park et al*.* [5–8] when applied to templated PNs written by pre-clerkship medical students for an OSCE. This rubric has been validated in a similar exercise for senior students modeled after the USMLE Step 2 CS exam but ours is the first description of its use in a pre-clerkship student population.

Compared to findings of from previous studies applying the UIC-COM Scoring Rubric to senior students, our pre-clerkship students’ section and composite scores were lower, especially on the Differential Diagnosis, Justification, and Workup sections. There are several possible explanations for this difference. First, because only pre-clerkship students completed this OSCE, we do not know how senior students would have performed on the same note-writing task, and therefore cannot rule out that the cases were poorly constructed. However, we think this is unlikely given the rigor of the case writing process to which we adhered. Students had more trouble with one of the cases (case B), as evidenced by the lower scores and completion rates. We believe this is because case B has more pertinent positives and negatives in the history, requires more organ systems to be investigated, and has a disconnect between the history and physical (given the SP’s inability to imitate certain findings).

Next, many students found the 10-min time limit to be a major factor contributing to the difficulty of the templated PN. While this was the same time allotment afforded to senior students in previous studies, it may have been too short for these junior students. We found diminishing completion rates with each subsequent section of the templated PN. Because the Documentation section comes first, students may have spent too much time writing their history and physical exam and then run out of time to complete the subsequent sections. In addition, because the templated PN-writing task was a formative assessment, the pre-clerkship students may not have expended as much effort as the senior students, for whom passing the OSCE was a requirement for graduation.

Further, students may have lacked adequate training in written communication skills to write a problem-focused PN after having spent 45 min taking a complete history and physical with the SP. Their pre-clerkship doctoring course mostly emphasizes history-taking and physical exam skills, and they have had less exposure to written and verbal communication of differential diagnoses and treatment plans.

But ultimately, even after accounting for the caveats described above, the pre-clerkship students’ worse performance compared to senior students on the templated PN-writing task may be attributable to less mature clinical reasoning skills. Perhaps at this early stage of training, students are so focused on obtaining a comprehensive and accurate history and performing the physical exam correctly that they lack the cognitive bandwidth to synthesize the data they have collected into a differential diagnosis, especially when under a time limit. This is supported by the higher scores on the Documentation section than on the Differential Diagnosis and Justification sections. (Students may have scored higher on the Workup section despite lower completion rates because they have some familiarity with the diagnostic tests used for various organ systems and could have guessed the right answers.) They may have seen an insufficient number of cases to have developed illness scripts for the most common chief complaints. Or maybe pre-clerkship students require the Socratic method to refine their hypotheses and are unprepared to present a differential diagnosis without immediate feedback from a teacher. While there are many possible reasons why early students are too inexperienced for this exercise, it is premature to dispense with it entirely before making the modifications we discuss below (especially increasing the time limit).

The work we present here was a pilot study, and due to the differences between our population and the one in which the UIC-COM Scoring Rubric was validated, it is perhaps unsurprising that measures of internal consistency reliability for the section scores on both cases were poor. However, this was also found in the above referenced multisite study. In our study, item discrimination for some sections was high but Cronbach’s alpha was low for both cases [10]. The low Cronbach’s alpha can be explained partly by the small number of items (four) and by task specificity, yet it may also be traced to the time limit and students’ lack of experience, as discussed previously. Cronbach’s alpha may have been especially low for case B due to its content (see above). Inter-rater reliability in our two-grader analysis was fair, suggesting that more rater training is needed.

In spite of these challenges, most students felt that the templated PN-writing exercise was useful and appropriate to their level of training according the post-OSCE survey. Knowing that they had to write the templated PN—which could have led students to focus more on remembering the details of the history and physical rather than on communicating effectively with the SP—did not detract from their interactions with the SP. A plurality of students preferred the templated PN to the open-ended PN, and half thought the two note types were equally difficult to write. Though many medical schools teach their pre-clerkship students to write long-form notes and then pare back as they gain clinical acumen, some students may benefit from a more structured, streamlined approach to help organize their clinical reasoning.

Our study has several strengths. We employed a validated rubric for scoring templated PNs to students at a different stage of the learning process. Note scoring was fairly efficient, and we believe senior students, residents, or fellows could serve as graders where faculty are not available. Indeed, recent studies have shown that even non-clinicians can be trained to score patient notes effectively [11, 12]. Moreover, we provided students formative feedback based on their performance. We achieved a high response rate on our post-OSCE survey, in which students rated the templated PN-writing experience positively. There were also some limitations. Ours was a single-institution study with a small sample size, as YSM has a small class size (approximately 100 students per class year), and we only analyzed data from one class year. The video interviewing of SPs and examination of mannequins rather than live SPs could have impacted students’ performance, either negatively or positively. Although substantial case specificity has been shown with the use of the UIC-COM PN Scoring Rubric, each student wrote a templated PN for only one of two cases, so we were unable to conduct a generalizability study [5–8].

We plan to use the lessons learned from the 2020 pre-clerkship OSCE to improve the rubric’s validity in subsequent years. (1) In future iterations, students will complete the same templated PN-writing exercise without a strict time limit, but with a suggested limit of 15 min. (2) We will record how much time students actually spend to guide modifications to the note-writing task. (3) We are replacing case B with a new case where the history and physical exam are concordant. (4) We will also compare students’ templated and open-ended PNs for the same case to determine if both instruments identify the same students as needing remediation. In addition, comparing the templated and open-ended PNs will allow us to observe how students’ clinical reasoning evolves as they spend more time considering a case and assimilating outside resources. (5) We will consider increasing the number of cases and having multiple graders score the templated PNs. (6) We may prompt the students to conduct a problem-focused, rather than comprehensive, history and physical, for at least one of the cases. (7) We may add another survey to be completed after the students receive feedback on their templated PNs to assess their response to the feedback. (8) When COVID-19 restrictions ease, we plan to return to a fully in-person OSCE, although the standardized video interview remains an appealing alternative when in-person assessment is not possible [13]. (9) We may incorporate the templated PN-writing exercise into multiple OSCEs across the curriculum to track students’ performance over time.

## Conclusions

In summary, we found that pre-clerkship medical students performed poorly on a templated PN-writing task in an OSCE when scored with a validated rubric. We feel that this difference in performance is at least partly due to underdeveloped clinical reasoning skills in the junior students. Reliability was suboptimal, likely due to the students’ inexperience and the strict time limit. Students rated the exercise positively. Developing validated assessments of clinical reasoning is pressing now that the USMLE Step 2 CS exam has been eliminated, and we plan to use the lessons described herein to improve our pre-clerkship OSCE for the future.

## Supplementary Information

**Additional file 1: Appendix A. **Exemplar Note for Case A. **Appendix B. **Exemplar Note for Case B. **Appendix C.** Post-OSCE Survey

## Data Availability

The datasets used and analyzed during the current study are available from the corresponding author on reasonable request.
