# Supplementary material for: Applying a validated scoring rubric to pre-clerkship medical students’ standardized patient notes: a pilot study
Source: BMC Med Educ. 2023 Jul 13;23:504. doi: 10.1186/s12909-023-04424-9 (PMC10339528; doi:10.1186/s12909-023-04424-9)
Supplement: Supplementary file 1 — Additional file 1: Appendix A. Exemplar Note for Case A. Appendix B. Exemplar Note for Case B. Appendix C. Post-OSCE Survey [file 12909_2023_4424_MOESM1_ESM.docx]

**Appendix A: Exemplar Note for Case A**

**CC:** Headache

**HISTORY:**

HPI: Patient presents with headaches x 2 weeks. Whole head hurts, feels like a dull pain/pressure, 2/10 intensity. Headaches start in the morning, last several hours. Getting worse every day. Improved with Advil. Vomited upon awaking last 2 days, no preceding nausea. Never had headaches before. No preceding trauma. No fatigue, weight loss, fevers/chills, vision changes, photophobia, phonophobia, rhinorrhea, sore throat, focal weakness/numbness, dizziness/lightheadedness, abdominal pain, diarrhea, constipation.

PMH: HLD

PSH: none

Meds: Atorvastatin 10 mg daily, Advil PRN

Allergies: PCN (hives/throat swelling)

SH: non-smoker, 2-3 drinks EtOH/month, no drugs. Supportive family & friends, no acute stressors or financial concerns.

FH: No history of brain tumor or aneurysm, no cancer.

**PHYSICAL EXAMINATION:**

VS: (within normal limits)

General: Well-appearing, in no acute distress.

HEENT: No scalp or temporal artery tenderness. No TMJ tenderness. No sinus tenderness. PERRLA. No papilledema. EOMs intact. Mucous membranes moist, oropharynx clear. Neck supple. No cervical lymphadenopathy.

Neuro:

MSE: Awake and alert, oriented x 3.

CNs: II-XII intact.

Sensory: Grossly intact to light touch bilaterally.

Motor: Normal bulk and tone. Power 5/5 throughout.

Reflexes: DTRs 2+ throughout, Babinski negative, no ankle clonus.

Coordination: Finger-nose-finger intact bilaterally.

Gait: Normal.

**DATA INTERPRETATION:**

#1 Brain tumor

- 1. Supporting history: New-onset headache in patient without history of migraine, holocephalic, signs of increased intracranial pressure (worse in the morning, associated with vomiting without nausea)
  2. Supporting physical: N/A

OR

#1 Idiopathic intracranial hypertension (IIH) / pseudotumor cerebri

- 1. Supporting history: New-onset headache in patient without history of migraine, holocephalic, signs of increased intracranial pressure (worse in the morning, associated with vomiting without nausea)
  2. Supporting physical: N/A

#2 or 3 Migraine

1. Supporting history: Gradual onset, lasting several hours, alleviated with NSAID, absence of vision changes and systemic symptoms (fevers/chills, weight loss).
2. Supporting physical: Normal neurologic exam, no papilledema.

OR

#2 or 3 Tension headache

1. Supporting history: Gradual onset, lasting several hours, alleviated with NSAID, absence of vision changes and systemic symptoms (fevers/chills, weight loss).
2. Supporting physical: Normal neurologic exam, no papilledema.

**DIAGNOSTIC STUDIES:**

1. MRI brain w/ and w/o contrast

**Appendix B: Exemplar Note for Case B**

**CC:** Shortness of breath

**HISTORY:**

HPI: Patient reports dyspnea on exertion x 2 weeks. Can’t walk more than 100 yards. Previously could walk 1-2 miles without stopping. After starts walking feels short of breath for 5-10 minutes, improves with rest. Associated with bilateral ankle swelling and 2-pillow orthopnea and PND. MD prescribed Lasix 20 mg by phone yesterday, took 2 doses, had large urine output, symptoms improved significantly. No dizziness/lightheadedness, chest pain, palpitations, cough, urinary changes, abdominal pain, diarrhea/constipation, heat/cold intolerance.

PMH: HTN, hypothyroidism

PSH: N/A

Meds: Lasix 20 mg PRN, levothyroxine 112 mcg daily, lisinopril 20 mg daily, amlodipine 5 mg daily

Allergies: None

SH: non-smoker, 4-5 EtOH drinks/year, no drugs

FH: Mother has HLD, father had HTN and died of MI at age 80. Brother has HLD.

**PHYSICAL EXAMINATION:**

VS: (within normal limits)

General: Well-appearing, in no acute distress.

HEENT: No thyromegaly, thyroid tenderness, or palpable thyroid nodules. JVP 5 cm.

Chest: Clear to auscultation bilaterally, no dullness to percussion.

Heart: Regular rate and rhythm, normal S1/S2, no murmurs/rubs/gallops. PMI non-displaced.

Abdomen: Abdomen soft, non-tender, non-distended, no palpable masses or organomegaly, BS normoactive in all 4 quadrants.

Extremities: Warm and well-perfused, no edema, DP and PT pulses 2+ bilaterally.

**DATA INTERPRETATION:**

#1 Congestive heart failure

- 1. Supporting history: Dyspnea on exertion, bilateral ankle edema, orthopnea, PND, improvement with Lasix, history of HTN, family history of CAD.
  2. Supporting physical: N/A

#2 ACS or stable angina

- 1. Supporting history: Dyspnea on exertion, history of HTN, family history of CAD.
  2. Supporting physical: N/A

#3 Nephrotic syndrome

1. Supporting history: Dyspnea on exertion, bilateral ankle edema, improvement with Lasix.
2. Supporting physical: N/A

OR

#3 Hypothyroidism

- 1. Supporting history: Dyspnea on exertion, bilateral ankle edema, history of hypothyroidism.
  2. Supporting physical: N/A

**DIAGNOSTIC STUDIES:**

1. EKG
2. Chest X-ray
3. Basic metabolic panel
4. Urinalysis
5. TSH
6. TTE (+/- POC)

**Maybe**

BNP

Troponin

**NO**

LHC

Stress test

**Appendix C: Post-OSCE Survey**

I found writing the USMLE-style templated note to be useful for:

|  | Strongly agree | Somewhat agree | Neither agree nor disagree | Somewhat disagree | Strongly disagree |
| --- | --- | --- | --- | --- | --- |
| Writing an H&P |  |  |  |  |  |
| Developing a differential diagnosis |  |  |  |  |  |
| Justifying my differential diagnosis |  |  |  |  |  |
| Devising a treatment plan |  |  |  |  |  |

What was the **level of difficulty** of writing the USMLE-style templated note **compared to** writing an open-ended note?

- More difficult than an open-ended note
- About the same level of difficulty
- Less difficult than an open-ended note

Display This Question:

If What was the level of difficulty of writing the USMLE-style templated note compared to writing an... = More difficult than an open-ended note

What made it more difficult?

________________________________________________________________

________________________________________________________________

________________________________________________________________

________________________________________________________________

________________________________________________________________

What is your **preference level** in writing the USMLE-style templated note **compared to** writing an open-ended note?

- I prefer writing the templated note
- I do not prefer one type of note over the other
- I prefer writing the open-ended note

|  |  |
| --- | --- |

You indicated that *_________*. Please explain your answer.

________________________________________________________________

________________________________________________________________

________________________________________________________________

________________________________________________________________

________________________________________________________________

Knowing that I had to write a templated note negatively affected my ability to engage with the standardized patient.

- Strongly agree
- Somewhat agree
- Neither agree nor disagree
- Somewhat disagree
- Strongly disagree

The encounter with the patient over the video screen was a good opportunity to:

|  | Strongly agree | Somewhat agree | Neither agree nor disagree | Somewhat disagree | Strongly disagree |
| --- | --- | --- | --- | --- | --- |
| Practice a medical history |  |  |  |  |  |
| Practice interpersonal skills |  |  |  |  |  |
| Receive feedback on my clinical skills |  |  |  |  |  |

Some medical schools are having students **narrate the steps in a physical exam** rather than demonstrate them on a mannequin. Would you have preferred this? Why do you feel this way?

- Yes ________________________________________________
- No ________________________________________________

Rate your agreement with the following statements.

|  | Strongly agree | Somewhat agree | Neither agree nor disagree | Somewhat disagree | Strongly disagree |
| --- | --- | --- | --- | --- | --- |
| The encounter with the mannequin was a good opportunity to practice physical examination. |  |  |  |  |  |
| The P-OSCE was appropriate for my level of training. |  |  |  |  |  |
| Given the circumstances, the P-OSCE was a formative learning experience. |  |  |  |  |  |

What other feedback, comments, or suggestions do you have about the P-OSCE?

________________________________________________________________

________________________________________________________________

________________________________________________________________

________________________________________________________________

________________________________________________________________
